# Ribonuclease RNase Z is an evolutionarily conserved deAMPylase

**DOI:** 10.1073/pnas.2515155122

**Published:** 2025-11-20

**Authors:** Meghomukta Mukherjee, Alex Pon, Timea Goldberg, Krzysztof Pawłowski, Anju Sreelatha

**Affiliations:** ^a^Department of Physiology, University of Texas Southwestern Medical Center, Dallas, TX 75390; ^b^Department of Molecular Biology, University of Texas Southwestern Medical Center, Dallas, TX 75390; ^c^HHMI, University of Texas Southwestern Medical Center, Dallas, TX 75390; ^d^Charles and Jane Pak Center for Mineral Metabolism and Clinical Research, University of Texas Southwestern Medical Center, Dallas, TX 75390

**Keywords:** AMPylation, selenoprotein O, RNase BN, Endonuclease, adenylylation

## Abstract

Protein AMPylation is a highly conserved posttranslational modification in which adenosine monophosphate (AMP) is covalently attached to protein substrates. Our studies revealed that the mitochondrial AMPylase, Selenoprotein O (SelO), regulates cellular metabolism and oxidative stress response through AMPylation of key metabolic enzymes. Remarkably, SelO-mediated AMPylation is conserved in bacteria and humans, yet the enzyme that removes the AMP from modified proteins remains unknown. We show that the ribonuclease, RNase Z, is both necessary and sufficient to catalyze deAMPylation of AMPylated substrates. These results establish RNase Z as a moonlighting enzyme with previously unrecognized functional roles beyond tRNA processing, expanding our understanding of its biological significance. Furthermore, identification of an evolutionarily conserved deAMPylase highlights the importance of reversible AMPylation as a biological regulatory mechanism, akin to well-studied post translational modifications such as protein phosphorylation.

## Significance

Selenoprotein O (SelO) is a conserved AMPylase found in bacteria and humans, underscoring its fundamental role in cellular regulation. While AMPylases act as writer enzymes by attaching AMP to proteins, the identity of the corresponding eraser enzymes or deAMPylases remained elusive. Here, we identify RNase Z, an evolutionarily conserved endoribonuclease known for its role in tRNA maturation, as a deAMPylase that removes AMP from SelO modified substrates. Our findings expand the functional repertoire of RNase Z by demonstrating its dual functionality in RNA processing and posttranslational protein modification.

Selenoprotein O (SelO) encodes a pseudokinase domain that is conserved from bacteria to humans (1). Structural studies have revealed that SelO harbors unique active site residues that facilitate the binding of ATP in an inverted orientation in comparison to canonical kinases. Through this unconventional mode of binding, SelO catalyzes AMPylation, rather than phosphorylation, of multiple proteins involved in metabolism and redox homeostasis to protect cells from oxidative damage and cell death (2). This cytoprotective role of SelO suggests broader implications for its function in pathological conditions, including cancer. Notably, elevated mRNA levels of SelO are associated with higher mortality in patients with metastatic melanoma. SelO AMPylates key metabolic enzymes, including aconitase and succinate dehydrogenase, and promotes melanoma metastasis in an immunocompetent murine model (3). These studies highlight the importance of mitochondrial AMPylation as a signaling mechanism in both cellular stress responses and cancer progression.

Collectively, there are three protein folds that catalyze AMPylation: nucleotidyltransferase, FIC (filamentation induced by cAMP), and pseudokinase (4–6). In line with the signaling analogy, if AMPylases serve as writer molecules, the erasers or deAMPylases catalyze the removal of AMP known as deAMPylation (7). For example, the intracellular bacterial pathogen *Legionella pneumophila* utilizes reversible AMPylation for temporal regulation of Rab1 activity to establish a replicative niche during infection (8, 9). SidM AMPylates Rab1 in the initial phase of the infection (8). Rab1-AMP is subsequently deAMPylated by SidD, which harbors a protein phosphatase fold (9, 10). The nucleotidyltransferase domain of GlnE and the FIC domain of FicD have been shown to act bifunctionally as both AMPylases and deAMPylases (11–13). GlnE contains two nucleotidyltransferase domains connected by a regulatory domain that is responsive to the cellular nitrogen status (14). Although the two nucleotidyltransferase domains are structurally similar, amino acid residues in the active sites promote opposite reactions such that the N-terminal domain catalyzes deAMPylation and the C-terminal domain catalyzes AMPylation (14). Importantly, the only known deAMPylase in mammalian cells is FicD, that localizes to the luminal side of the endoplasmic reticulum (15). The most recently identified AMPylase, SelO, is conserved in bacteria, yeast, and humans and localizes to the mitochondria in eukaryotes (1, 2). However, the enzyme(s) that catalyzes deAMPylation of SelO substrates are unknown.

Based on the three folds that catalyze deAMPylation (nucleotidyltransferase, FIC, and phosphatase), we sought to identify the enzyme that catalyzes deAMPylation of SelO substrates (*SI Appendix*, Fig. S1 *A* and *B*). There are relatively few human enzymes with the protein nucleotidyltransferase fold and only a single FIC protein (5, 16, 17). In contrast, protein phosphatases which are part of the hydrolase superfamily, are one of the largest protein classes known to catalyze the cleavage of covalent bonds in proteins and small molecules (18).

Here, we report RNase Z, a member of the metallo-hydrolase superfamily, as a potent deAMPylase of AMPylated SelO substrates. RNase Z is a widely conserved enzyme that cleaves the 3’ trailer sequence of precursor tRNAs to generate mature tRNAs (19–21). Surprisingly, deletion of RNase Z in *Escherichia coli* is viable and did not alter precursor tRNA processing or global protein translation (22–24). These studies along with the low efficiency of RNase Z-mediated tRNA cleavage alluded to the possibility of alternative activities for RNase Z (25–27). We reveal the dual activity of RNase Z which catalyzes deAMPylation of SelO substrates, in addition to tRNA processing which is much less efficient. Furthermore, we utilized a recently developed AMPylation enrichment strategy to demonstrate that deletion of RNase Z increases levels of AMPylated proteins in cells (28). Thus, our studies uncover RNase Z as a moonlighting protein with deAMPylase activity.

## Biochemical Analysis of Potential deAMPylases Identified Through a Bioinformatics Screen

In *E. coli*, GlnE and SelO are the only known enzymes that catalyze AMPylation. Notably, the enzymes responsible for the deAMPylation of SelO substrates remain elusive. To identify the enzymes that catalyze deAMPylation, we generated a list of candidate proteins that meet three criteria. First, we performed FFAS sensitive sequence searches to identify human proteins that belong to the nucleotidyltransferase, phosphatase, phosphodiesterase, and metallo-hydrolase superfamilies of enzymes (29). Considering four distinct superfamilies for the bioinformatics screen, including the metallo-hydrolases, ensures that potentially novel or poorly characterized enzymes are not excluded from the analysis. Based on the evolutionary conservation of SelO, we sorted for proteins that are conserved in *E. coli*, yeast, and humans, using BLAST searches for homologs. Finally, we used Uniprot annotations and the Mitominer and DeepLoc servers to search for enzymes that localize to the mitochondria in eukaryotes (30, 31). Our analysis generated a list of candidate proteins based on our criteria for enzyme fold, conservation, and localization (Datasets S1 and S2).

**Fig. 1.**
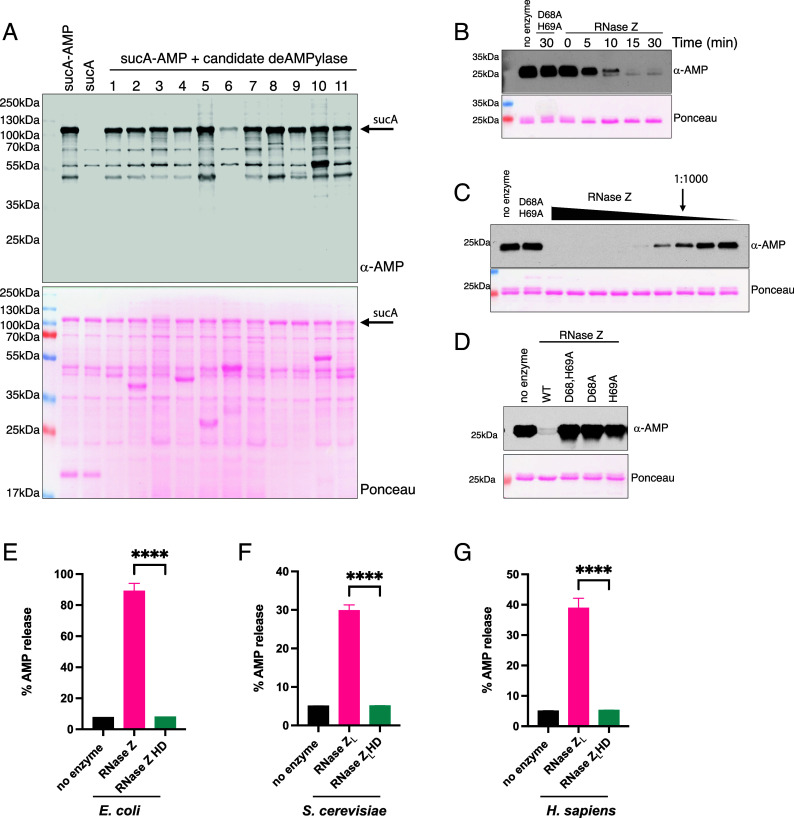
RNase Z catalyzes deAMPylation of protein substrates. (*A*) Protein immunoblots of *Escherichia coli* lysates expressing sucA or sucA-AMP in the absence or presence of candidate deAMPylases. His-SUMO tagged candidate proteins correspond to the following gene names: 1. gloB, 2. gloC, 3. mgtA, 4. nagD, 5. rsfS, 6. rbn, 7. pcnB, 8. CCA1, 9. YMR130W, 10. YKR070W, and 11. YBR242W (See Dataset S1 for additional information). The ponceau stained membrane depicting protein expression of the candidates is shown. (*B*) Protein immunoblots of time-dependent deAMPylation of 2 μM sodA-AMP by 11 nM RNase Z but not the inactive D68A, H69A mutant. (*C*) Protein immunoblots of concentration-dependent deAMPylation of sodA-AMP by RNase Z but not the inactive D68A, H69A mutant. The arrow denotes reaction containing 4 nM RNase Z: 4 μM sodA-AMP (1:1,000). Concentration of RNase Z ranged from 426 nM to 1 nM. (*D*) Protein immunoblots of in vitro deAMPylation assay of 4 μM sodA-AMP by 0.4 μM RNase Z WT but not the inactive mutants. (*E*) Representative bar graph depicting AMP release from AMPylated peptides incubated with *E. coli* RNase Z or in the inactive mutant (RNase Z HD) measured using AMP-Glo™ assay. Data reported as % AMP release from the total AMPylated peptides [TITSS-Y(AMP)-YR] per reaction. Results are representative of 3 independent experiments. *P < 0.0001. (*F*) Representative bar graph depicting AMP release from AMPylated peptides incubated with *S. cerevisiae* RNase Z_L_ or in the inactive mutant (RNase Z_L_ HD) measured using AMP-Glo^TM^ assay. Data reported as % AMP release from the total AMPylated peptides [EVYRGAE-Y(AMP)-AVDG] per reaction. Results are representative of 3 independent experiments. *P < 0.0001. (*G*) Representative bar graph depicting AMP release from AMPylated peptides incubated with *H. sapiens* RNase Z_L_ or in the inactive mutant (RNase Z_L_ HD) measured using AMP-Glo™ assay. Data reported as % AMP release from the total AMPylated peptides [Ac-SEYVP-T(AMP)-VFDNYGC-NH_2_] per reaction. Results are representative of 3 independent experiments. *P < 0.0001.

To screen for deAMPylation activity, we coexpressed the candidate deAMPylases in *E. coli* with a known substrate of SelO, sucA, the E1 component of the bacterial α-ketoglutarate dehydrogenase complex (2) Protein immunoblotting of cell lysates demonstrates that AMPylated sucA (sucA-AMP) is detected by the anti-AMP antibody while unmodified sucA is not (Fig. 1*A*). Expression of *E. coli* RNase Z reduced AMPylation of sucA in cells (Fig. 1*A*, candidate 6). RNase Z (RNase BN, rbn) belongs to the metallo-β-lactamase fold hydrolase superfamily which contains proteins known to have diverse catalytic activities including RNA processing and the hydrolysis of small molecules. RNase Z is a conserved endoribonuclease that cleaves the 3’ trailer sequence of precursor tRNAs to generate mature tRNAs.

## RNase Z Catalyzes deAMPylation of Protein Substrates

To determine whether RNase Z catalyzes deAMPylation in vitro, we incubated recombinant *E. coli* RNase Z with AMPylated superoxide dismutase (sodA-AMP) and analyzed deAMPylation by protein immunoblotting and mass spectrometry. We observed time and concentration-dependent deAMPylation of sodA-AMP (Fig. 1 *B* and *C*). Notably, RNase Z catalyzed potent deAMPylation of sodA-AMP at a concentration 1,000-fold lower than the substrate (Fig. 1*C*). Furthermore, mutation of the active site metal binding HxHxDH motif abolished deAMPylation activity (Fig. 1*D*). Intact mass analysis of sodA revealed a mass decrease of ~329 Daltons corresponding to the loss of AMP after incubation with wild-type *E. coli* RNase Z (WT), but not the inactive mutant (HD) (*SI Appendix*, Fig. S2). Thus, RNase Z catalyzes deAMPylation in vitro and in cells.

The crystal structure of tRNA bound *B. subtilis* RNase Z (PDB: 4GCW) depicts a functional dimer arranged head-to-head where the active site is positioned between the homodimers (*SI Appendix*, Fig. S3 *A* and *B*). The monomers are oriented in opposite directions, and a protruding flexible arm (known as the exosite) from one subunit binds and positions the tRNA in the active site of the opposing subunit for cleavage (32–34). To gain insight into the mechanism and the key residues that catalyze deAMPylation, we tested the necessity of the exosite for deAMPylation. Deletion of the exosite reduced deAMPylation activity of *E. coli* RNase Z (*SI Appendix*, Fig. S3*C*). These data suggest that conserved amino acid residues in the active site and substrate binding motif are required for efficient deAMPylation.

RNase Z is an evolutionarily conserved ribonuclease that plays a role in the maturation of tRNAs (35). In contrast to bacteria, eukaryotes have a long form RNase Z_L_ and a short form RNase Z_S_ that predominantly localize to the mitochondria and cytosol, respectively (36–38). Mitochondrial RNase Z_L_ is specific to eukaryotes and harbors two tandem RNase Z domains (35). The N-terminal domain lacks the catalytic HxHxDH motif but has an exosite for tRNA binding while the C terminal domain lacks the exosite but has the catalytic motif (*SI Appendix*, Fig. S4) (39). Thus, RNase Z_L_ consists of two linked RNase Z domains in the same polypeptide to function like the bacterial RNase Z dimer (39, 40). *E. coli* RNase Z, *S. cerevisiae* RNase Z_L_, and *H. sapiens* RNase Z_L_, but not the catalytic mutants (HD), released AMP upon incubation with AMPylated peptide substrates (Fig. 1 *E*–*G*). Collectively, these results suggest that deAMPylation is an evolutionarily conserved function of RNase Z.

## RNase Z Cleaves Precursor tRNA

**Fig. 2.**
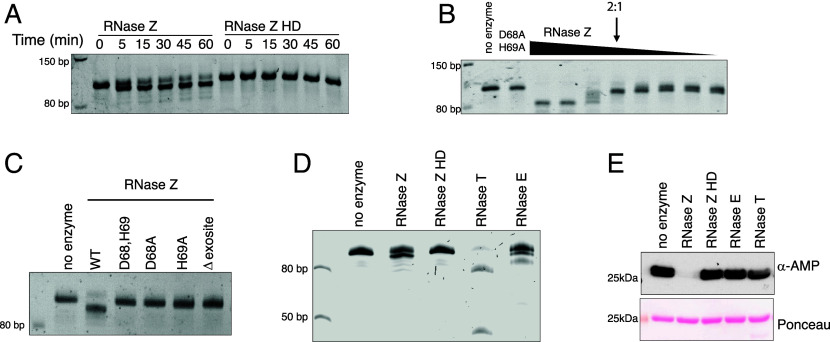
RNase Z cleaves precursor tRNA^PheV^. (*A*) Urea-PAGE analysis of time-dependent 0.1 μM pre-tRNA^PheV^ cleavage by 0.2 μM RNase Z but not the inactive D68A, H69A mutant. tRNA is visualized using SYBR™-gold nuclei acid stain. (*B*) Urea-PAGE analysis of concentration-dependent pre-tRNA^PheV^ cleavage by RNase Z but not the inactive D68A, H69A mutant. tRNA is visualized using SYBR™-gold nuclei acid stain. The arrow denotes reaction containing 0.2 μM RNase Z: 0.1 μM pre-tRNA^PheV^ (2:1). Concentration of RNase Z ranged from 1.4 μM to 0.0125 μM. (*C*) Urea-PAGE analysis of 0.1 μM pre-tRNA^PheV^ cleavage by 0.2 μM RNase Z but not the inactive mutants. (*D*) Urea-PAGE analysis of 0.06 μM pre-tRNA^PheV^ cleavage by RNase Z (0.25 μM), the endoribonuclease RNase E (0.5 μM), or the exoribonuclease RNase T (0.5 μM). tRNA is visualized using SYBR™-gold nuclei acid stain. (*E*) Protein immunoblots of in vitro deAMPylation assay of 4 μM sodA-AMP by 0.4 μM RNase Z, 0.8 μM RNase E, or 4 μM RNase T.

Given that RNase Z is known to catalyze the removal of 3’ nucleotides of precursor tRNA, we tested the ribonuclease activity of recombinant *E. coli* RNase Z with *E. coli* precursor tRNA, pre-tRNA^PheV^ (19, 26). RNase Z catalyzed the cleavage of pre-tRNA^PheV^ in a time and concentration-dependent manner (Fig. 2 *A* and *B*). Notably, the catalytic activity required a 2-fold molar excess of enzyme to pre-tRNA^PheV^ substrate for visualization of the cleaved mature tRNA, in agreement with previously published reports (24, 26, 41). Mutation of the active site amino acids and the substrate binding motif eliminated tRNA cleavage, similar to our observation with RNase Z deAMPylation activity (Fig. 2*C*). Collectively, these results suggest that the amino acid residues in the active site of RNase Z facilitate deAMPylation and ribonuclease activity.

To determine whether deAMPylation activity is a general property of endo or exoribonucleases, we tested the activity of the 3’ exonuclease RNase T and endonuclease RNase E. RNase E and RNase T are active ribonucleases as observed with the cleavage of pre-tRNA^PheV^ (Fig. 2*D*). However, neither catalyze deAMPylation in vitro (Fig. 2*E*). Furthermore, coexpression of RNase Z, but not RNase E or RNase T, catalyzed deAMPylation of sucA-AMP in cells (*SI Appendix*, Fig. S5*A*). Thus, the dual activity of deAMPylation and tRNA cleavage appears to be unique to RNase Z and not a general property of ribonucleases.

## RNase Z Is Specific for SelO-Mediated AMPylation

**Fig. 3.**
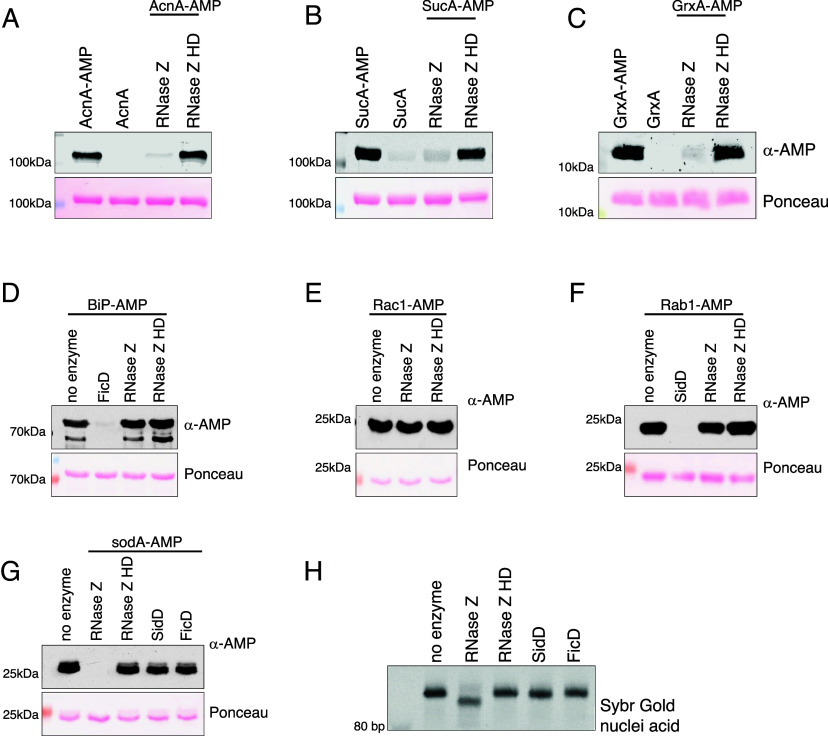
RNase Z is specific for SelO-mediated AMPylation. Protein immunoblotting of in vitro deAMPylation assay of (*A*) acnA-AMP, (*B*) sucA-AMP, (*C*) grxA-AMP by RNase Z, or RNase Z HD. (*D*) Protein immunoblots of in vitro deAMPylation assay of BiP-AMP by RNase Z, RNase Z HD, or FicD. (*E*) Protein immunoblots of in vitro deAMPylation assay of Rac1-AMP by RNase Z, or RNase Z HD. (*F*) Protein immunoblots of in vitro deAMPylation assay of Rab1-AMP by RNase Z, RNase Z HD, or SidD. (*G*) Protein immunoblots of in vitro deAMPylation assay of sodA-AMP by RNase Z, RNase Z HD, FicD, or SidD. (*H*) Urea-PAGE analysis of pre-tRNA^PheV^ cleavage by RNase Z, RNase Z HD, FicD, or SidD. tRNA is visualized using SYBR™-gold nuclei acid stain.

The pseudokinase domain of SelO catalyzes the AMPylation of multiple substrates involved in redox homeostasis and metabolism (2). Unlike SelO, most of the other known AMPylases, GlnE, FicD, and SidM, all have a single substrate. The broad substrate specificity of SelO is poorly understood but not unexpected given that kinases often have multiple substrates. Interestingly, RNase Z catalyzed the deAMPylation of several substrates of SelO in addition to sodA, including aconitase (acnA), glutaredoxin (grxA), and sucA in vitro (Fig. 3 *A*–*C*). Furthermore, coexpression of RNase Z WT, but not the inactive HD mutant, reduced SelO-mediated AMPylation in cells (*SI Appendix*, Fig. S5*B*). Although RNase Z catalyzed the deAMPylation of all tested SelO substrates, the efficiency of deAMPylation varied among substrates in a concentration and time-dependent manner (*SI Appendix*, Fig. S5 *C* and *D*).

Next, we tested whether *E. coli* RNase Z can act on AMPylated proteins that are not substrates of SelO, such as BiP-AMP, Rab1-AMP, and Rac1-AMP (6). RNase Z did not deAMPylate BiP-AMP, Rab1-AMP, or Rac1-AMP in contrast to their cognate deAMPylases (Fig. 3 *D*-*F* and *SI Appendix*, Fig. S1). Furthermore, other deAMPylases do not act on SelO substrates (Fig. 3*G*). These findings agree with previous reports of specificity of the *Legionella* deAMPylase SidD, which deAMPylates Rab1-AMP but not the Rho GTPases, Rac1-AMP, or Cdc42-AMP (42). Thus, the deAMPylation activity of RNase Z is specific for SelO substrates akin to other deAMPylases. To further assess the dual activity of RNase Z in comparison to other deAMPylases, we tested whether the known deAMPylases can act as RNases. The bifunctional enzyme, FicD, and the phosphatase fold enzyme, SidD, did not catalyze cleavage of precursor tRNA (Fig. 3*H*). These results emphasize the dual characteristic of RNase Z as a deAMPylase as well as a ribonuclease.

## Deletion of RNase Z Increases AMPylation in Cells

To date, GlnA and SelO substrates are the only reported AMPylated proteins in *E.coli* (7). While AMPylated GlnA is readily detectable in cell lysates, SelO mediated AMPylation occurs at much lower levels (28). Due to the low abundance of AMPylated proteins, the hyperactive mutant, SelO V242A, is required to enrich and detect SelO-mediated AMPylation in *E. coli* when SelO is expressed under the endogenous promoter (*SI Appendix*, Fig. S6*A*).

**Fig. 4.**
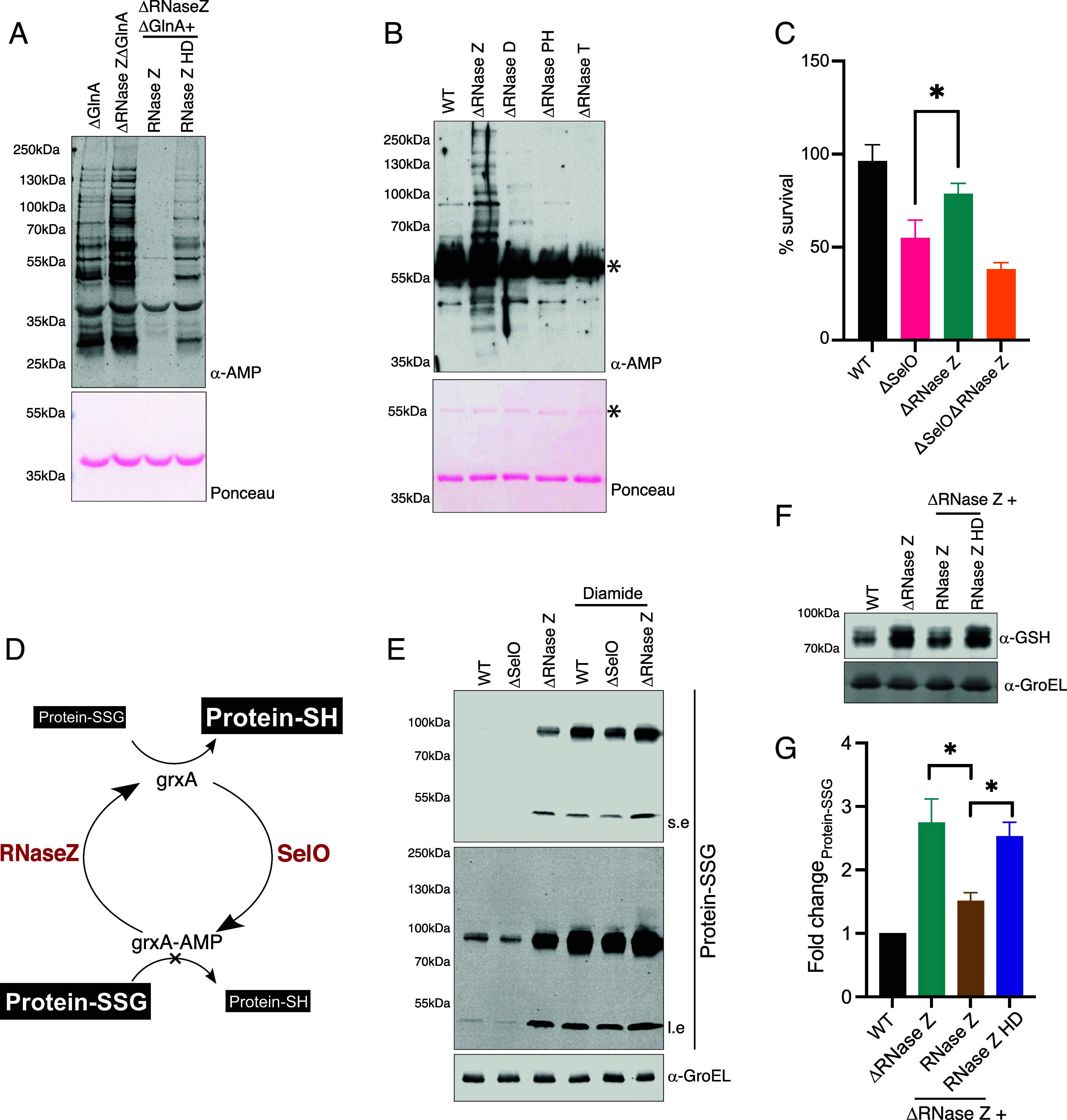
Deletion of RNase Z increases AMPylation in cells. (*A*) Protein immunoblots of AMPylated proteins enriched using GST-hinT H101N from *E. coli* BW25113 GlnA knockout (ΔGlnA), RNase Z and GlnA double knockout (ΔRNase Z ΔGlnA), double knockout (ΔRNase Z ΔGlnA) expressing RNase Z-flag, or RNase Z-flag HD. All strains are expressing SelO V242A under the endogenous SelO promoter. (*B*) Protein immunoblots of AMPylated proteins enriched using GST-hinT H101N from *E. coli* BW25113 wildtype (WT), RNase Z knockout, RNase D knockout, RNase PH knockout, or RNase T knockout. All strains are expressing SelO V242A with the endogenous promoter. *denotes AMPylated GlnA. (*C*) Percent survival of *E. coli* WT, SelO knockout (ΔSelO), RNase Z knockout (ΔRNase Z), or double knockout (ΔSelO ΔRNase Z) following treatment with pyocyanin. Results are representative of 3 independent experiments. *P < 0.05. (*D*) Glutaredoxins catalyze deglutathionylation of glutathionylated protein (protein-SSG) to regenerate the free protein thiol (protein-SH). SelO AMPylates glutaredoxins (grxA) to inhibit deglutathionylation while RNase Z catalyzes deAMPylation of grxA. Reversible protein AMPylation of grxA by SelO and RNase Z may thus regulate protein glutathionylation. (*E*) Protein immunoblots of *E. coli* extracts obtained from WT, SelO knockout (ΔSelO), or RNase Z knockout (ΔRNase Z), treated with diamide or water control. Extracts were probed for glutathionylation (protein-SSG) and GroEL (loading control). Short exposure (s.e) and long exposure (l.e) for α-glutathionylation immunoblotting are depicted. (*F*) Representative protein immunoblots of *E. coli* extracts obtained from WT, RNase Z knockout (ΔRNase Z), RNase Z knockout expressing RNase Z (ΔRNase Z + RNase Z), or RNase Z knockout expressing RNase Z HD (ΔRNase Z + RNase Z HD) treated with diamide. Extracts were probed for glutathionylation (α-GSH) and GroEL (loading control). (*G*) Densitometric quantification of protein glutathionylation (protein-SSG) levels from representative immunoblots, as depicted in (*F*), and normalized to wild type control. *P < 0.05.

To determine the physiological importance of RNase Z, we first examined whether deletion of RNase Z increases the cellular levels of SelO-mediated AMPylation. RNase Z knockout *E. coli* displayed more AMPylated proteins compared to wild type *E. coli* (Fig. 4*A* and *SI Appendix*, Fig. S6B). Deletion of GlnA enhanced the visibility of SelO-mediated AMPylation (Fig. 4*A*). Consistent with our in vitro deAMPylation assays, RNase Z deAMPylates SelO substrates but did not act on non-SelO substrates such as GlnA (noted with * in Fig. 4*B* and *SI Appendix*, Fig. S6B). GlnA therefore provides a reliable internal control to verify equal sample loading in the AMPylation immunoblots. Expression of RNase Z, but not the inactive mutant, rescued the deAMPylation defect in RNase Z knockout *E. coli* (Fig. 4*A* and *SI Appendix*, Fig. S6B). Although RNase Z was expressed from its native promoter on a plasmid to complement the knockout strain, its expression levels were higher than endogenous (*SI Appendix*, Fig. S6*C*). We hypothesize that this increased expression may result in residual catalytic activity, consistent with previous reports of residual RNase activity in *B. subtilis* H65A mutant (43).

Next, we tested whether deletion of other RNases such as RNase D, RNase PH, and RNase T affect deAMPylation. In agreement with our hypothesis that deAMPylation is unique to RNase Z, deletion of the nonessential RNases did not alter AMPylation levels compared to WT *E. coli*. Consistently, RNase Z knockout *E. coli* have increased AMPylation compared to wild type or deletion of RNase D, RNase PH, or RNase T (Fig. 4*B*). Thus, RNase Z is necessary and sufficient to catalyze deAMPylation of SelO substrates in *E. coli*.

SelO AMPylates proteins that have well-defined roles in oxidative stress response and protects cells from oxidative damage and cell death (2). Therefore, we hypothesized that deletion of RNase Z would increase protein AMPylation and protect cells from oxidative stress. In agreement with this hypothesis, we observed lower survival in SelO knockout *E. coli* in comparison to wild type or RNase Z knockout *E. coli* upon treatment with ROS inducer, pyocyanin (Fig. 4*C*). SelO adds an AMP group while RNase Z removes it, exerting opposite effects on protein AMPylation. Because AMPylation is a prerequisite for deAMPylation, deletion of both SelO and RNase Z would be predicted to phenocopy the deletion of SelO. Consistent with our hypothesis, we observed reduced survival in the double knockout cells akin to SelO knockout (Fig. 4*C* and *SI Appendix*, Fig. S6*D*).

Under conditions of oxidative stress, conjugation of a glutathione moiety protects protein thiols from over oxidation in a post translational modification known as protein S-glutathionylation. Glutaredoxins remove the glutathione moiety from protein thiols to return them to the native state (44). SelO AMPylates glutaredoxin (grxA) to inhibit deglutathionylation during oxidative stress and therefore protects protein thiols from oxidative damage (Fig. 4*D*) (2). In agreement with previous studies, SelO knockout *E. coli* have lower levels of glutathionylated proteins in comparison to wild type *E. coli* as detected by antibodies against S-glutathionylation (Fig. 4*E*) (2). Importantly, RNase Z knockout *E. coli* demonstrated increased glutathionylation corresponding with the increased AMPylation levels observed in cells (Fig. 4 *A* and *E*). Addition of a thiol oxidizing agent diamide, which is known to promote the formation of protein-glutathione adducts, revealed a marked decrease in protein glutathionylation in the SelO knockout *E. coli* in comparison to the wild type and RNase deficient *E. coli* (Fig. 4*E*). *E. coli* lacking RNase Z and SelO demonstrated decreased protein glutathionylation similar to SelO deficient *E. coli* (*SI Appendix*, Fig. S6*E*). Furthermore, the increased glutathionylation observed in RNase Z deficient *E. coli* was fully complemented by wildtype (WT) RNase Z but not the catalytically inactive mutant (Fig. 4 *F* and *G*). Collectively, these results suggest that reversible AMPylation can regulate global protein glutathionylation levels in bacteria.

## Discussion

We identified RNase Z as a potent deAMPylase of SelO substrates. Previous studies have shown that RNase Z plays a role in the maturation of tRNAs by cleaving the 3’-end of precursor tRNAs. Interestingly, RNase Z is a more efficient deAMPylase compared to ribonuclease in vitro as observed by the enzyme: substrate molar ratio required for RNase (2:1) and deAMPylation (1:1,000) activity. Furthermore, mutational studies suggest that deAMPylation and endonuclease activity are catalyzed by the same amino acids in the active site. We analyzed other tRNA 3’-end processing RNases for deAMPylation activity and demonstrate that the dual activity is specific to RNase Z. Importantly, RNase Z knockout *E. coli* have increased levels of SelO-mediated AMPylation. Deletion of SelO decreases protein S-glutathionylation while deletion of RNase Z increases protein S-glutathionylation, suggesting that reversible AMPylation is a regulatory mechanism in oxidative stress response.

RNase Z belongs to the metallo-β-lactamase superfamily which hydrolyzes diverse substrates including β-lactam antibiotics, DNA, and RNA (45). The structures of *E. coli* and *B. subtilis* RNase Z depict monomers composed of the αβ/βα sandwich fold that are oriented in opposite directions, with a long flexible exosite extending away from the active site (32, 33, 43, 46). The exosite orients the tRNA for cleavage while the catalytic Asp68 acts as a general base to deprotonate water, which then acts as a nucleophile on the phosphate of tRNA to cleave the phosphodiester bond (47). A similar mechanism of catalysis is observed with the deAMPylase, SidD, in which the binuclear bridging water is deprotonated by the catalytic aspartates to cleave the adenyl-O-tyrosyl linkage of Rab1-AMP to produce Rab1 and AMP (10, 42). Similarly, we propose that the catalytic Asp68 acts as a general base for RNase Z to catalyze hydrolytic deAMPylation based on the conserved metallohydrolase fold, active site mutagenesis studies, and AMP release. The biochemical similarity of deAMPylation and RNase activity is exemplified by their shared mechanism of phosphodiester bond cleavage (20, 42). Given that AMP is a building block of RNA, the ability of RNase Z to act on both tRNA and AMPylated protein substrates raises intriguing questions about the evolutionary and mechanistic versatility of this enzyme.

Eukaryotes have a cytosolic short form (RNase Z_S_) and a long form (RNase Z_L_) which exhibits dual localization to the nucleus and mitochondria (36–38). RNase Z_L_, encoded by ELAC2, is specific to eukaryotes and harbors two tandem RNase Z domains (36, 37). ELAC2 consists of an N-terminal pseudo-RNase Z domain (NTD) which lacks the catalytic HxHxDH motif, and C-terminal RNase Z domain (CTD) connected by a linker (39, 40). Unlike *E. coli* RNase Z, ELAC2 is essential in humans and is linked to increased prostate cancer susceptibility (39). Cardiomyopathy-associated mutations in ELAC2 resulted in tRNA processing defects and impaired translation (48, 49). Prostate cancer related mutations, S217L, A541T, and R781H, identified by genetic linkage analysis do not affect RNase activity in vitro (50) (51). A mechanistic understanding of the RNase and deAMPylation activities of ELAC2 may pave the way for therapeutic strategies.

Our data demonstrate the dual functionality of RNase Z; however, the mechanisms that regulate these activities in cells remain unclear. One possibility is that specific interaction partners may bias RNase Z toward one activity over the other, resulting in context-dependent functional outcomes. A recent report demonstrated that ELAC2 requires binding to a protein complex consisting of MRPP1 (TRMT10C) and MRPP2 (SDR5C1), which act as processing platforms, to efficiently cleave mitochondrial tRNA (52, 53). The deAMPylation and RNase activity of ELAC2 in cells may be regulated by binding of the MRPP1/MRPP2 complex or orientation of the pseudo-RNase Z domain (NTD) with the C-terminal RNase Z domain (CTD). Given the increasing complexity of eukaryotic signaling, the deAMPylation activity of ELAC2 in the mitochondria warrants further investigation.

Protein AMPylation is relatively understudied compared to other PTMs, such as phosphorylation, and thus signifies high potential for discovering new biology and regulatory mechanisms. Here, we identify a previously undocumented function for RNase Z as a protein deAMPylase, revealing unexpected enzymatic versatility beyond its canonical role in tRNA processing. These findings establish reversible AMPylation as a fundamental and conserved biological regulatory mechanism catalyzed by distinct enzymes.

## Materials and Methods

### Reagents and Bacterial Strains.

Glutathione agarose was purchased from Fisher Scientific (PI16101). Mouse anti-AMP antibodies (clone IDs B992601, B992602, and B992603) were purchased from Biointron based on (54). *E. coli* BW25113 and knockout strains of RNase Z (JW2263-1), SelO (JW1696-1), RNase D (JW1793-1), RNase T (JW1644-1), RNase PH (JW3618-2), and GlnA (JW3841-1) were obtained from the *E. coli* genetic stock center (CGSC) at Yale. *E. coli* double knockout strain of RNase Z and GlnA (BW25113 ΔRNase Z ΔGlnA) and RNase Z and SelO (BW25113 ΔRNase Z ΔSelO) was constructed using the λ-Red recombinase system as previously described (55). Briefly, the pKD46 plasmid was electroporated into BW25113 ΔGlnA or BW25113 ΔSelO and maintained at 30 °C. Expression of recombinase was induced with 100 mM L-arabinose and electrocompetent cells were prepared. PCR product containing chloramphenicol cassette flanked by ~50 bp homology arms corresponding to the 5’ and 3’ of RNase Z gene were generated using pKD3 as template. The purified PCR product was electroporated into recombinase expressing BW25113 ΔGlnA cells or BW25113 ΔSelO. Recombinants were selected on chloramphenicol containing plates and verified by colony PCR and immunoblotting with polyclonal rabbit α-RNase Z antibodies.

### Generation of Constructs.

*E. coli* SelO ppSumo, *E. coli* SelO D348A ppSumo, *E. coli* SelO V242A ppSumo, and *E. coli* hinT H101N pGEX-rTEV were generated in (2, 28). Complementation constructs were generated using the pBAD expression plasmid. *E. coli* promoter-Myc-SelO pBad MycHis A (referred to as SelO pBad for simplicity) was generated by cloning the *E. coli* SelO coding sequence with an N-terminal Myc tag and 1000 basepairs upstream harboring the endogenous SelO promoter into pBad MycHis A. Subsequently, *E. coli* RNase Z coding sequence with a C terminal Flag tag and 1000 basepairs upstream was cloned into *E. coli* promoter-Myc-SelO pBad MycHis A. The resulting plasmids were introduced into the knockout strains to restore expression of the corresponding protein under the endogenous promoter. Arabinose was not added to induce expression, as complementation relied on the endogenous promoter to achieve near physiological expression levels and prevent overexpression from the pBAD promoter.

Plasmids for the coexpression of *E. coli* substrates (sucA, grxA, sodA, or acnA) and *E. coli* SelO in pETDuet were generated as described in (2). Human Rab1a was cloned into MCS1 (multiple cloning site 1) of petDuet1 in-frame with an N-terminal 6X His tag. Subsequently, *L. pneumophila* SidM was cloned without a tag into MCS2. *E. coli* RNase Z, RNase E, and RNase T coding sequence were amplified by PCR using DH5a genomic DNA as template. The amplified open reading frames were cloned into ppSumo or pProEx expression vectors (2). *E. coli* RNase Z exosite deletion was generated by deleting amino acids 153-203, as defined in (56), using site directed mutagenesis with Agilent PfuTurbo DNA polymerase (*SI Appendix*, Table S1). *S. cerevisiae* RNase Z was amplified from BY4741 genomic DNA. *H. sapiens* RNase Z was amplified from Ultimate ORF Lite human cDNA collection (Life technologies NM_018127.6). *S. cerevisiae* RNase Z and *H. sapiens* RNase Z were cloned into ppSumo expression vector.

### Protein Expression and Purification.

AMPylated sodA was generated for in vitro deAMPylation assays by coexpressing *E. coli* SelO ppSumo and sodA pProEX in Rosetta DE3 cells. Cells were grown in LB broth supplemented with 100 μg/mL ampicillin and 50 μg/mL kanamycin to OD_600_ of 0.6 to 0.8. Protein expression was induced with 0.4 mM IPTG for 16 to 18 h at 22 °C. Cells were harvested by centrifugation and lysed in 50 mM Tris- HCl pH 8, 300 mM NaCl, 1 mM PMSF, and 1 mM DTT by sonication. Lysate was clarified by centrifugation at 25,000 × *g* for 25 min. The soluble lysate was incubated with Ni-NTA beads for one hour at 4 °C. Beads were passed through a column and washed with 20 column volumes of 50 mM Tris-HCl pH 8, 300 mM NaCl, 10 mM imidazole, and 1 mM DTT. The protein was eluted with 50 mM Tris-HCl pH 8, 300 mM NaCl, 300 mM imidazole, 1 mM DTT. The eluted protein was then buffer exchanged to 25 mM Tris pH 7.5, 1 mM DTT. The AMPylated sodA protein was further purified using a EnrichQ ion exchange column attached to a BioRad NGC chromatography system.

*E. coli* RNase Z ppSumo, *E. coli* RNase Z pProEx, RNase E ppSumo, or RNase T ppSumo were transformed into BL21 cells. *S. cerevisiae* RNase Z or *H. sapiens* RNase Z were transformed into Rosetta pLysS. Cells were grown in LB broth supplemented with 100 μg/mL ampicillin or 50 μg/mL kanamycin to OD_600_ of ~0.8. Protein expression was induced with 0.4 mM IPTG for 16 to 18 h at 22 °C. Cells were harvested by centrifugation and lysed in 50 mM Tris- HCl pH 8, 300 mM NaCl, 1 mM PMSF, and 1 mM DTT by sonication. Lysate was clarified by centrifugation at 25,000 x *g* for 25 min and supernatant was incubated with Ni-NTA beads for one hour at 4 °C. Samples were passed over a column, and beads were washed with 20 CV of 50 mM Tris-HCl pH 8, 300 mM NaCl, 10 mM imidazole, and 1 mM DTT. Proteins were eluted with 5 CV of 50 mM Tris-HCl pH 8, 300 mM NaCl, 300 mM imidazole, 1 mM DTT. 6xHis-SUMO tag was removed by cleavage with ULP1 at 4 °C overnight for RNase E, RNase T, *S. cerevisiae* RNase Z and *H. sapiens* RNase Z. The proteins were then separated by size exclusion chromatography using the HiLoad 16/600 Superdex 200 column attached to a BioRad NGC chromatography system pre-equilibrated with 10 mM Tris-HCl pH 8, 150 mM NaCl, and 2 mM DTT.

AMPylated human BiP (coexpressed with Fic E247G and purified) and AMPylated Rac (coexpressed with VopS and purified) was generously gifted by the Orth lab (13, 57). petDuet-Rab1:SidM, as well as plasmids for SelO substrates (petDuet-sucA:SelO, petDuet-acnA:SelO, petDuet-grxA:SelO) were transformed in Rosetta ΔSelO cells. To generate the non-AMPylated controls, SelO substrates were cloned into MCS1 with inactive SelO D256A in MCS2 of petDuet. Cells were grown in LB supplemented with 100 μg/mL ampicillin to OD600 ~ 0.8 and induced with 0.4 mM IPTG overnight at 22 °C. Cells were harvested by centrifugation and lysed in 50 mM Tris- HCl pH 8, 300 mM NaCl, 1 mM PMSF, and 1 mM DTT by sonication. Lysate was clarified by centrifugation at 25,000 x *g* for 25 min and supernatant was incubated with Ni-NTA beads for one hour at 4 °C. Samples were passed over a column, and beads were washed with 20 CV of 50 mM Tris-HCl pH 8, 300 mM NaCl, 10 mM imidazole, and 1 mM DTT. Proteins were eluted with 5 CV of 50 mM Tris-HCl pH 8, 300 mM NaCl, 300 mM imidazole, 1 mM DTT. The proteins were then separated by size exclusion chromatography using HiLoad 16/600 Superdex 200 or Superdex 75 column attached to a BioRad NGC chromatography system pre-equilibrated with 10 mM Tris-HCl pH 8, 150 mM NaCl, and 2 mM DTT.

### GST- hinT H101N Enrichment.

#### Preparation of GST-hinT H101N bead resuspension.

For each reaction, 2 μg of GST-hinT H101N was nutated with 10 μl bed volume of glutathione beads in 10 mM Tris pH 8, 150 mM NaCl at 4 °C for approximately 1 h. Beads were centrifuged 5,000 x *g* for 30 s at 4 °C and washed twice with 10 mM Tris pH 8, 150 mM NaCl.

#### Lysate preparation and binding.

10 mL cultures of BW25113 or knockout strains(ΔRNase Z, ΔSelO, ΔRNase D, ΔRNase T, ΔRNase PH, ΔGlnA, ΔGlnA ΔRNase Z, ΔSelO ΔRNase Z) were grown in LB overnight at 37 °C. BW25113 WT or knockout strains complemented with *E. coli* SelO V242A pBad or *E. coli* SelO:RNase Z pBAD were grown in LB with 100 μg/mL ampicillin overnight at 37 °C. Cells were lysed in 10 mM Tris pH 8, 150 mM NaCl, 0.1% TX-100, 1 mM PMSF, 1 mM DTT by sonication. Lysates were centrifuged at 21,000 × *g* for 10 min at 4 °C. Supernatant was transferred and normalized to approximately 2 mg/mL. Approximately 2 mg of normalized lysates were nutated with the prepared GST-hinT H101N bead resuspension at 4 °C for 2 h or overnight. Beads were centrifuged 5,000 × *g* for 30 s at 4 °C and washed three times with 10 mM Tris pH 8, 150 mM NaCl, 0.1% TX-100, 1 mM DTT. Beads were resuspended in SDS loading buffer containing 1% β-mercaptoethanol and boiled. Samples were resolved by SDS-PAGE, transferred to nitrocellulose membranes and analyzed for protein AMPylation using anti-AMPylation (clone 1G11).

### Bioinformatics.

The Fold & Function Assignment System (FFAS) server (58) for remote protein sequence similarity detection was used to find candidates for deAMPylases of SelO substrates in humans. Thus, FFAS searches were performed for sequence similarities between human proteins and the following enzyme superfamilies (Clans in the nomenclature of the Pfam database): Phosphohydrolase/phosphodiesterase (Pfam ID: HD_PDEase, CL0237), Metallo-hydrolase/oxidoreductase (Metallo-HOrase, CL0381), Haloacid dehalogenase-like (HAD, CL0137), Alkaline phosphatase-like (Alk_phosphatase, CL0088), Nucleotidyltransferase (NTP_transf, CL0260). For cases of borderline sequence similarity, it was verified using the HHpred server (59).

Evolutionary conservation between human, yeast, and bacterial proteins was analyzed by BLAST searches. The mitochondrial localization of human and yeast proteins was assigned using the SwissProt/Uniprot annotations or predicted using the Mitominer and DeepLoc servers (30, 31).

For deAMPylase candidates, the criteria of enzymatic fold, evolutionary conservation, and mitochondrial localization in eukaryotes were applied. Additionally, active site conservation was evaluated for candidate deAMPylases using the Weblogo algorithm (60) after collecting homologs by BLAST searches and aligning them using the MAFFT method (61).

### In Vivo deAMPylase Screening.

Rosetta ΔSelO cells were transformed with petDuet-sucA:SelO or petDuet-sucA:SelO D348A and candidate deAMPylases cloned into ppSumo (Dataset S1). Cells were grown in LB supplemented with 100 μg/mL ampicillin and 50 μg/mL kanamycin to OD600 ~ 0.9 and induced with 0.4 mM IPTG overnight at 22 °C or 18 °C.

For screening deAMPylases, whole cell lysate was prepared by adding SDS loading dye to overnight cells and boiling for 10 min. Samples were resolved by SDS-PAGE, transferred to nitrocellulose, and immunoblotted with anti-AMP (17G6) antibody.

To analyze deAMPylation of substrates in cells, Rosetta ΔSelO cells were transformed with petDuet-sucA:SelO, petDuet-grxA:SelO, petDuet-acnA: SelO or petDuet-sodA:SelO and ppSumo, RNase Z ppSumo, or RNase Z HD ppSumo. Cells were grown in LB broth supplemented with 100 μg/mL ampicillin and 50 μg/mL kanamycin to OD_600_ of 0.6 to 0.8. Protein expression was induced with 0.4 mM IPTG for 16 to 18 h at 22 °C. Cells were harvested by centrifugation and lysed in 50 mM Tris- HCl pH 8, 300 mM NaCl, 1 mM PMSF, and 1 mM DTT by sonication. Lysate was clarified by centrifugation at 25,000 × *g* for 25 min. The soluble lysate was incubated with Ni-NTA beads for one hour at 4 °C. Proteins were batch purified and eluted with 50 mM Tris-HCl pH 8, 300 mM NaCl, 300 mM imidazole, 1 mM DTT. The eluted proteins were normalized and prepared for SDS-PAGE and immunoblotting with anti-AMP antibodies.

### In Vitro deAMPylation Assay.

Typically, deAMPylation reactions were incubated at 37 °C for various time points and stopped with the addition of 10 mM EDTA. SDS loading buffer containing 1% β-mercaptoethanol was added to the samples and boiled at 95 °C for 5 min. Samples were resolved by SDS-PAGE, transferred to nitrocellulose membranes, and analyzed for protein AMPylation using anti-AMPylation (clone 17G6).

To test deAMPylation activity of *E. coli* RNase Z in comparison to RNaseZ Δexosite, reaction mixtures containing 50 mM Tris-HCl pH 7.5, 5 mM MnCl_2_, and 5 mM DTT, 5 μM of *E. coli* SodA-AMP and increasing concentration 9 nM – 143 nM of SUMO tagged *E. coli* RNase Z or RNaseZ Δexosite, were incubated at 37 °C for 30 min. RNase Z was added at a maximum concentration of 143 nM, followed by twofold serial dilutions, resulting in lowest concentration of 9 nM.

Concentration-dependent deAMPylation assays were performed in a reaction mixture containing 20 mM HEPES pH 6.5, 2 mM MnCl_2_, 2 mM DTT, 4 μM of *E. coli* SodA-AMP, and 426 nM to 1 nM of SUMO tagged *E. coli* RNase Z at 37 °C for 30 min. To determine time dependence, reaction mixtures containing 20 mM HEPES pH 6.5, 2 mM MnCl_2_, 2 mM DTT, 2 μM of *E. coli* SodA-AMP, and 11 nM of SUMO tagged *E. coli* RNase Z were incubated at 37 °C for 0, 5, 10, 15, 30, 45, and 60 min.

The following assays were performed to examine deAMPylation of sodA-AMP by RNase Z, RNase E, RNaseT, SidD, and FicD. Reaction mixtures containing 20 mM Tris-HCl pH 7.5, 2 mM MnCl_2_, 2 mM DTT, 4 μM of *E. coli* SodA-AMP, 0.4 μM of SUMO tagged *E. coli* RNase Z, 1.7 μM RNase E, 4 μM RNase T, 3 μM SidD or 2 μM FicD, were incubated at 37 °C for 30 min.

The following assays were performed to determine the deAMPylation activity of RNase Z toward BiP-AMP or Rac1-AMP, and Rab1-AMP. Reaction mixtures containing 20 mM Tris-HCl pH 7.5, 2 mM MnCl_2_, 2 mM DTT, substrates (1 μM of BiP-AMP or 5 μM Rac1-AMP, or Rab1-AMP), and 1 μM enzymes (SUMO tagged *E. coli* RNase Z, FicD, or SidD) were incubated at 37 °C for 30 min.

DeAMPylation assays for RNase Z catalytic site mutants were performed in reaction mixtures containing 50 mM Tris-HCl pH 7.5, 5 mM MnCl_2_, and 5 mM DTT, 4 μM *E. coli* SodA-AMP, and 0.4 μM SUMO tagged *E. coli* RNase Z or mutants and incubated at 37 °C for 30 min. DeAMPylation assays for various substrates of SelO were performed in reaction mixtures containing 50 mM Tris-HCl pH 7.5, 5 mM MnCl_2_, and 5 mM DTT, 64 nM of *E. coli* RNase Z, and 1 μM acnA-AMP or sucA-AMP. For grxA-AMP deAMPylation assays, reaction mixtures contained 50 mM Tris-HCl pH 7.5, 5 mM MnCl_2_, and 5 mM DTT, 15 μM for grxA-AMP and 277 nM his tagged *E. coli* RNase Z.

### Western Blotting.

After the samples were resolved by SDS-PAGE and transferred to nitrocellulose membrane, total proteins were visualized with Ponceau S staining. The membranes were rinsed with TBST buffer (10 mM Tris pH 8, 150 mM NaCl, 0.1% Tween-20). The membranes were blocked with 5% milk in TBST for 1 h. The membranes were rinsed with TBST. The membranes were incubated with primary antibodies for 1 h at room temperature or overnight at 4 °C. Following incubation with primary antibodies, membranes were washed with TBST and incubated with secondary antibody for 1 h at room temperature. Membranes were washed with TBST and incubated with ECL western blotting substrate for detection using film or imaged with Licor Odyssey western blot imaging system.

### In Vitro Transcription.

Pre-tRNA^pheV^ petDuet was used as the template for PCR using Q5 high fidelity polymerase. PCR products were purified using Zymo DNA clean and concentrator kit and used as template for transcription using Ambion MAXIscript T7 in vitro transcription kit. Reactions were stopped with the addition of 25 mM EDTA, extracted with phenol:chloroform:isoamyl alcohol (25:24:1), and precipitated with 2.5 volumes of 95% ethanol for 2 h at −20 °C. Precipitated tRNA was washed with ice-cold 70% ethanol and air dried. Pellets were resuspended in nuclease free water and RNA concentration was estimated using Nanodrop 2000C.

### RNase Assays.

The RNase assays were incubated at 37 °C for 30 min (unless specified otherwise) and stopped with the addition of 10 mM EDTA followed by 2X RNA loading dye (95% Formamide, 0.02% SDS, 0.02% Bromophenol blue, 0.01% xylene cyanol, and 1 mM EDTA). The samples were boiled at 85 °C for 3 mins. The samples were resolved by Urea PAGE and detected by SYBR™ Gold staining.

For time-dependent RNase activity assay, 0.1 μM pre-tRNA^pheV^ was incubated with 0.2 μM SUMO tagged *E. coli* RNase Z in reaction mixture containing 20 mM HEPES-KOH pH 6.5, 2 mM MnCl_2_, and 2 mM DTT for 0, 5, 10, 15, 30, 45, or 60 min at 37 °C.

SUMO tagged *E. coli* RNase Z 1.4 μM to 0.0125 μM was used to determine concentration-dependent RNase activity at 37 °C for 60 mins in a reaction mixture containing 20 mM HEPES-KOH pH 6.5, 2 mM MnCl_2_, 2 mM DTT, 0.1 μM pre-tRNA^pheV^.

RNase assay with SidD and FicD were performed in reaction mixtures containing 20 mM HEPES-KOH pH 6.5, 2 mM MnCl_2_, 2 mM DTT, 0.08 μM pre-tRNA^pheV^, and 0.32 μM SidD, FicD, or RNase Z.

RNase assay with RNase Z catalytic and exosite mutants were performed in reaction mixtures containing 20 mM HEPES-KOH pH 6.5, 2 mM MnCl_2_, 2 mM DTT, 0.1 μM pre-tRNA^pheV^, and 0.2 μM RNase Z or mutants.

RNase assay with RNase E and RNase T were performed in reaction mixtures containing 20 mM HEPES-KOH pH 6.5, 2 mM MnCl_2_, 2 mM DTT, 0.0625 μM pre-tRNA^pheV^, and 0.25 μM RNase Z or 0.5 μM RNase E or 0.5 μM RNase T.

### AMP Release Assays.

AMP release for *E. coli* RNase Z was measured in a reaction mixture containing 20 mM Tris-HCl pH 7.5, 2 mM MnCl_2_, 1 mM DTT, 10 μM AMPylated peptides, and 2.5 μM *E. coli* RNase Z for 30 min at 37 °C. AMP release for *S. cerevisiae* and *H. sapiens* RNase Z_L_ were measured in a reaction mixture containing 20 mM HEPES pH 7.5, 2 mM MgCl_2_, 2 mM TCEP, 10 μM AMPylated peptides, and 10 μM *S. cerevisiae* and *H. sapiens* RNase Z_L_ for 60 min at 37 °C. The reactions were stopped with 2 mM EDTA. AMP peptides were a generous gift from Christian Hedberg (54). AMP release was measured using Promega AMP-Glo^TM^. The luminescence was measured using the SYNERGY-MX Biotek spectrometer and Gen03.02 microplate reader software.

### Bacterial Survival Assays.

*E. coli* BW25113 WT, BW25113 ΔSelO, BW25113 ΔRNase Z, BW25113 ΔSelO ΔRNase Z cells were grown for 16-18 h in LB broth at 37 °C. Stationary cultures were treated with 1 mg/mL pyocyanin or DMSO for 1 h at room temperature. Cell survival was estimated by plating 10^-6^ dilution of treated cells on LB agar plates. Survival was normalized to the number of colony forming units (cfu) in the DMSO control treatment. Results are representative of three independent experiments. % survival = (colony forming units in pyocyanin/colony forming units in DMSO control) x 100.

### Glutathionylation Assays.

*E. coli* BW25113 WT, BW25113 Δ SelO, BW25113 ΔRNase Z, BW25113 ΔSelO ΔRNase Z, or BW25113 ΔRNase Z complemented with *E. coli* SelO:RNase Z pBAD were grown in LB media for 16 to 18 h at 37 °C. Cells were washed and normalized to 1 OD/mL using 1× PBS and treated with 1 mM diamide for 10 min at room temperature. After treatment, 10 mM NEM was immediately added to cells and incubated at room temperature for 5 min. Cells were centrifuged 5,000 × *g* for 30 s and resuspended in SDS loading buffer with 2 mM NEM and without reducing agent. Samples were resolved by SDS-PAGE and transferred to nitrocellulose membranes. Protein S-glutathionylation was analyzed by immunoblotting using anti-glutathione mAB (Virogen 101-A).

### Generation of Polyclonal Antibodies.

Untagged *E. coli* SelO was purified as described previously (2). *E. coli* RNase Z pProEx was purified as described above. Recombinant proteins were then used to inoculate rabbits for the generation of rabbit antiserum (Cocalico Biologicals). IgG was partially purified by ammonium sulfate precipitation and further affinity purified using recombinant SelO or RNase Z coupled to HiTrap NHS-activated HP column (62, 63). Antibodies were aliquoted and stored at -80 °C. For the detection of *E. coli* RNase Z and SelO from BW25113 strains, western blotting was performed as described above with antibodies diluted 1:5000 in 2% milk.

## Supplementary Material

Appendix 01 (PDF)

Dataset S01 (XLSX)

Dataset S02 (XLSX)

## Data Availability

All study data are included in the article and/or supporting information.
