# Supplementary material for: Ribonuclease RNase Z is an evolutionarily conserved deAMPylase
Source: Proc Natl Acad Sci U S A. 2025 Nov 20;122(47):e2515155122. doi: 10.1073/pnas.2515155122 (PMC12663964; doi:10.1073/pnas.2515155122)
Supplement: Supplementary file 1 — Appendix 01 (PDF) [file pnas.2515155122.sapp.pdf]

Ribonuclease RNase Z is an evolutionarily conserved deAMPylase

Meghomukta Mukherjee[a], Alex Pon[a], Timea Goldberg[a], Krzysztof Pawłowski[b,c], and Anju Sreelatha [a,d,1] *

Supplemental information

Supplemental figures S1-S6.

Table S1: Oligonucleotides used in this study.

Dataset S1: Candidate list of deAMPylases identified using bioinformatic analysis.

Dataset S2: Expanded list of candidate deAMPylases

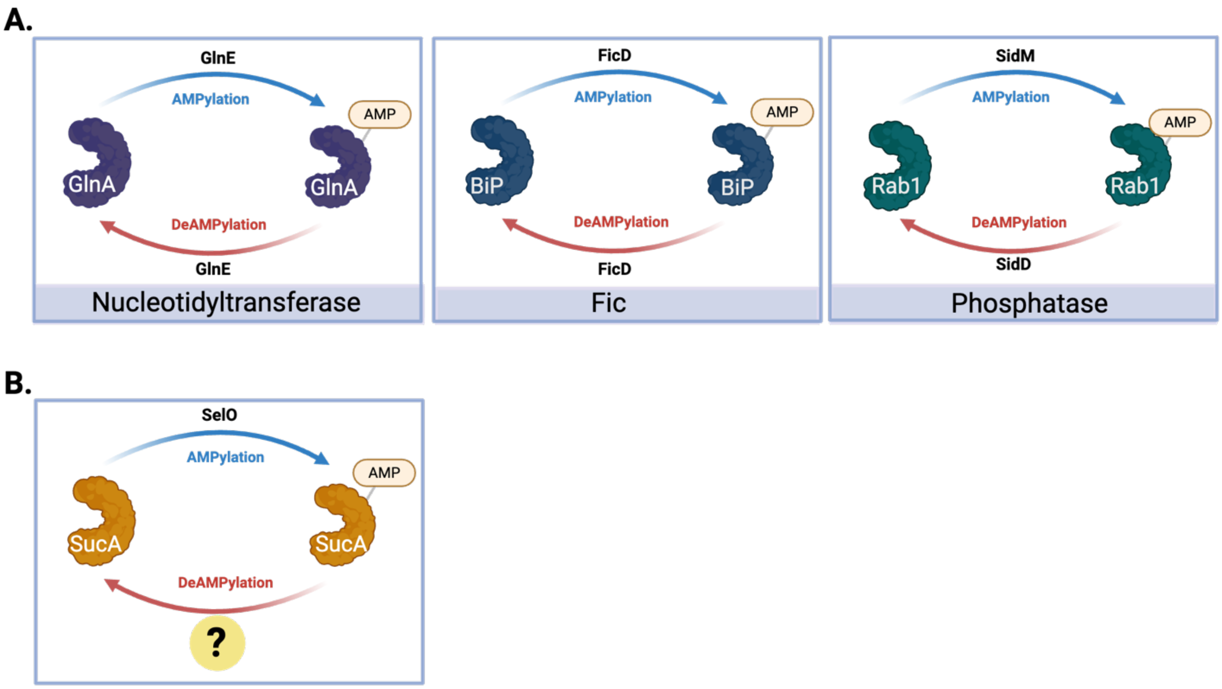

Figure S1. Schematic representation of the known AMPylases and deAMPylases.

(A) GlnE AMPylates and deAMPylates GlnA to regulate nitrogen assimilation in bacteria. FicD AMPylates and deAMPylates BiP to regulate unfolded protein response in metazoans. SidM AMPylates Rab1 while SidD deAMPylates Rab1-AMP to establish a replicative niche during *Legionella pneumophila* infection. The protein folds that catalyze deAMPylation such as nucleotidyltransferase, FIC, and phosphatase are highlighted below each panel.

(B) SelO catalyzes AMPylation of multiple substrates but the cognate deAMPylase is unknown. Created in BioRender. Sreelatha, A. (2025) https://BioRender.com/03ufjff

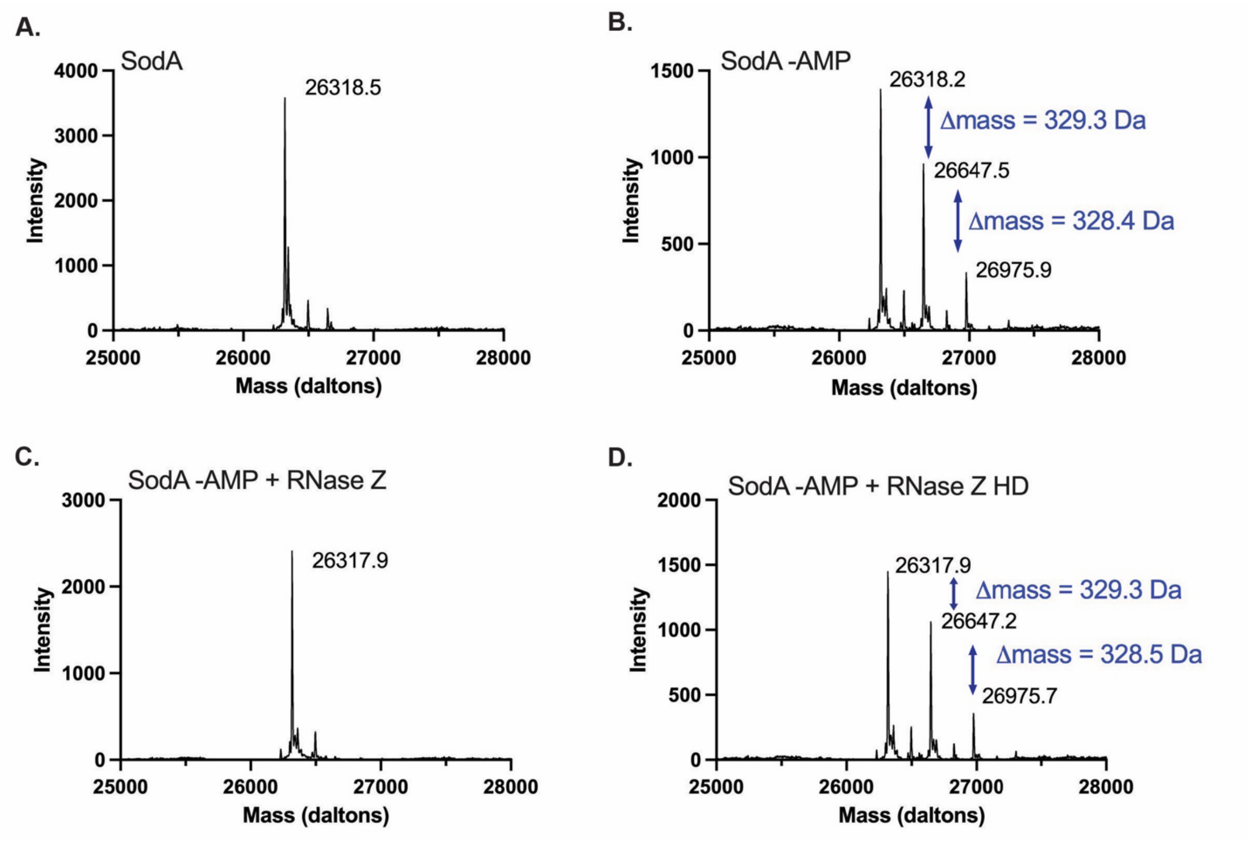

Figure S2. RNase Z deAMPylates sodA-AMP. Related to Figure 1

Intact mass LC/MS spectra of **(A)** sodA **(B)** sodA-AMP **(C)** sodA-AMP incubated with RNase Z, or **(D)** sodA-AMP incubated with RNase Z HD. Mass shift consistent with the addition of AMP (329 daltons) is noted in blue labels.

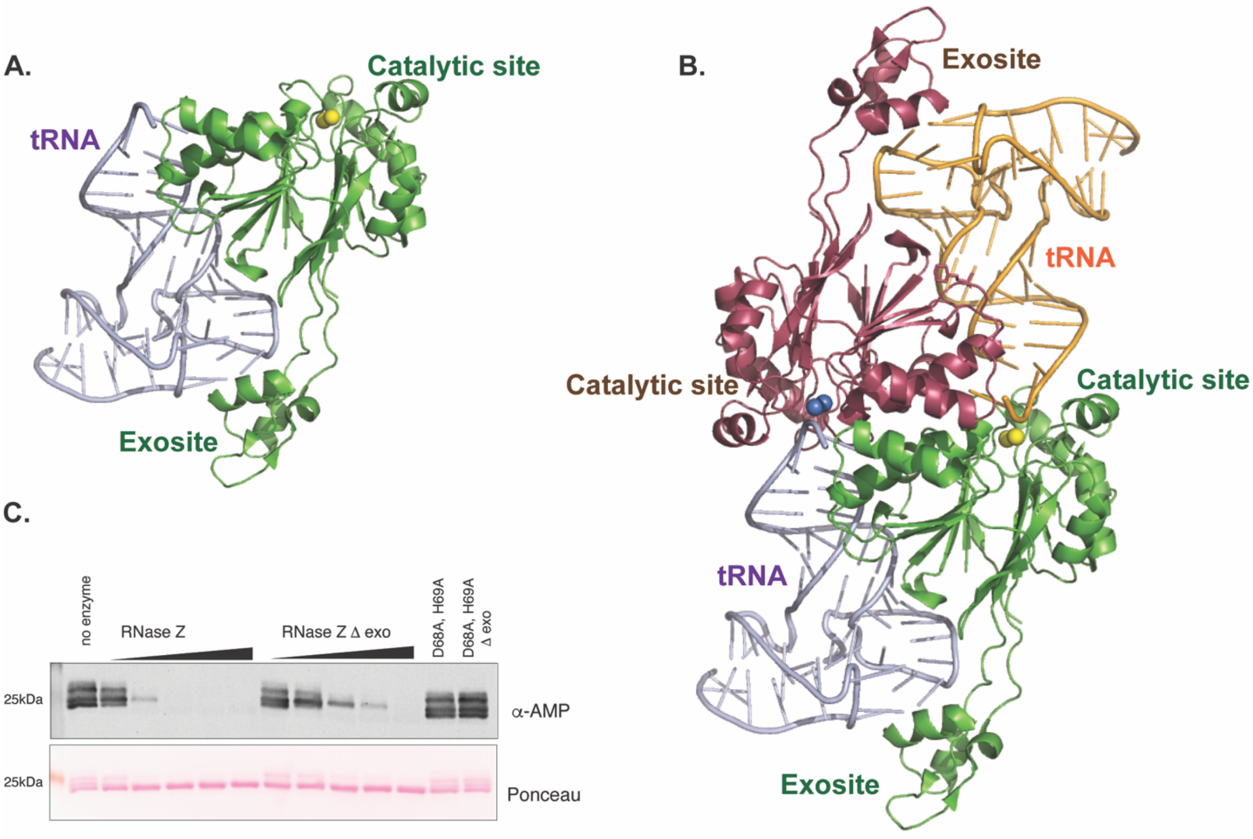

Figure S3. The exosite of RNase Z binds and positions tRNA for cleavage in the active site. Related to Figure 1.

(A) Ribbon representation of a monomer of RNase Z bound to tRNA (PDB 2CBN). tRNA shown in light purple, RNase Z depicted in green, zinc ions shown as yellow spheres.

(B) Ribbon representation of the dimeric RNase Z bound to tRNA. For a subunit of the dimer, tRNA shown in light purple, RNase Z depicted in green, zinc ions shown as yellow spheres. For the second subunit, tRNA shown in light orange, RNase Z depicted in maroon, zinc ions shown as blue spheres. tRNA from the first subunit is positioned for cleavage in the active site of the second subunit.

(C) Protein immunoblots of concentration-dependent deAMPylation of sodA-AMP by RNase Z or RNase Z lacking the exosite (RNase Z Δexo) but not the inactive D68A, H69A mutant. RNase Z was added at a maximum concentration of 143 nM, followed by two-fold serial dilutions, resulting in lowest concentration of 9 nM. The Ponceau stained membrane is shown.

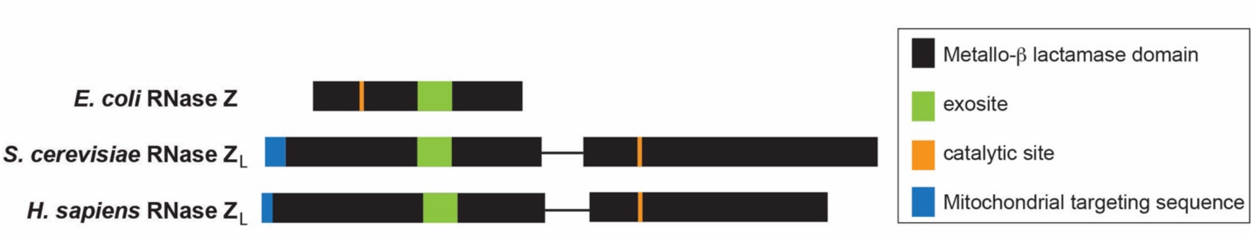

Figure S4. Human, yeast, and bacterial RNase Z catalyzes deAMPylation. Related to Figure 1

Schematic diagram of RNase Z homologues showing the predicted mitochondrial targeting sequence (MTS), metallo- β lactamase domain (MBL), exosite, and the catalytic HxHxDH motifs.

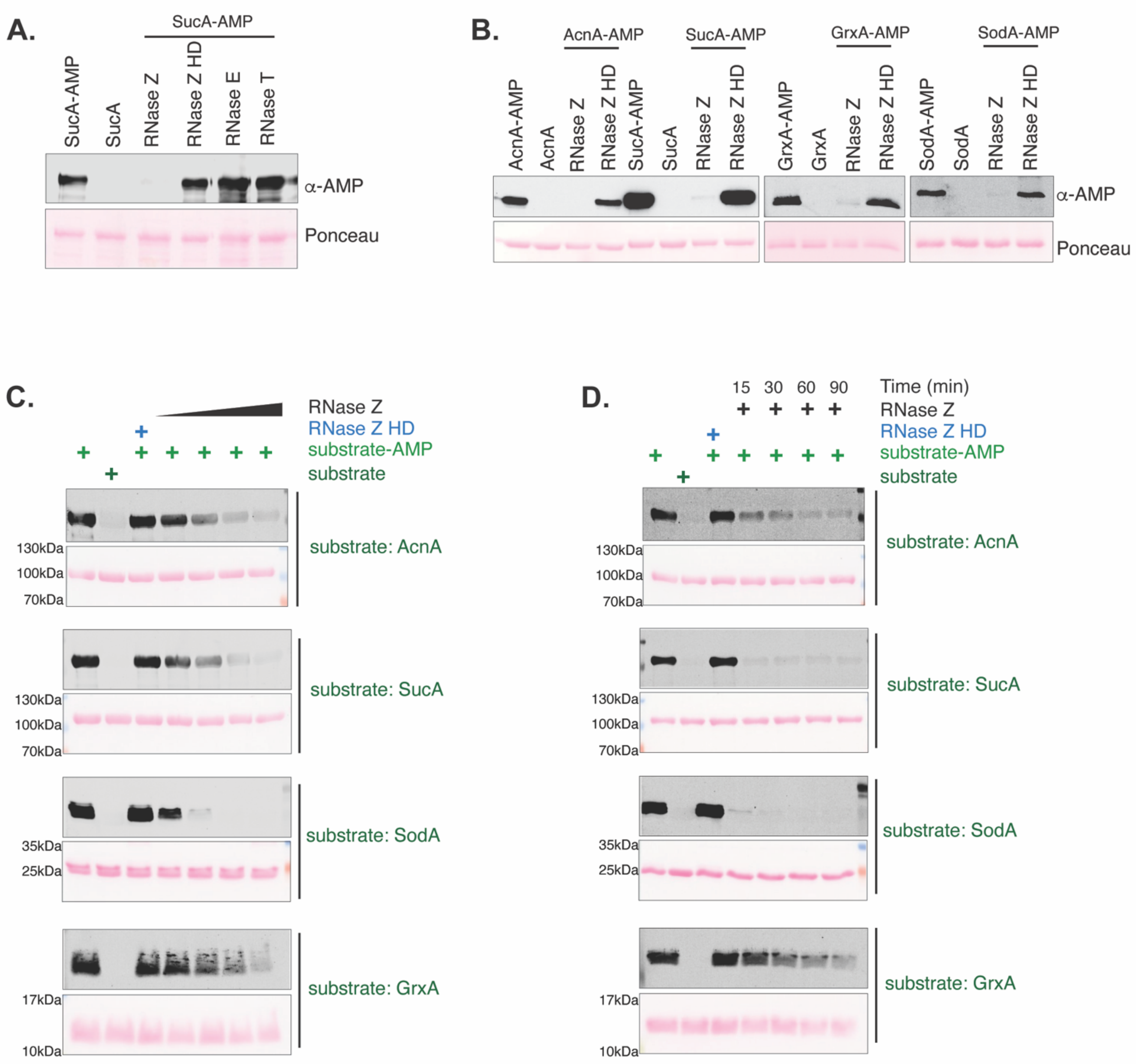

Figure S5. RNase Z deAMPylates multiple substrates of SelO in cells. Related to Figure 3.

(A) Protein immunoblotting of *E. coli* lysates expressing sucA or sucA-AMP with RNase Z, RNase E, or RNase T. The Ponceau stained membrane is shown.

(B) Protein immunoblotting of Ni-NTA affinity purified His-tagged proteins from *E. coli* co-expressing substrate proteins with RNase Z or RNase Z HD. The Ponceau stained membrane is shown.

(C) α-AMP protein immunoblotting of time-dependent *in vitro* deAMPylation of 1 μM acnA-AMP, 1 μM sucA-AMP, 5 μM sodA-AMP, or 20 μM grxA-AMP by 397 nM RNase Z but not the inactive D68A, H69A mutant. Reactions were incubated at 37°C for 15, 30, 60, or 90 minutes.

(D) α-AMP protein immunoblots of concentration-dependent deAMPylation of 1 μM acnA-AMP, 1 μM sucA-AMP, 5 μM sodA-AMP, or 20 μM grxA-AMP by RNase Z but not the inactive D68A, H69A mutant. RNase Z was added at a maximum concentration of 367 nM, followed by four-fold serial dilutions, resulting in lowest concentration of 6 nM. Reactions were incubated at 37°C for 60 minutes.

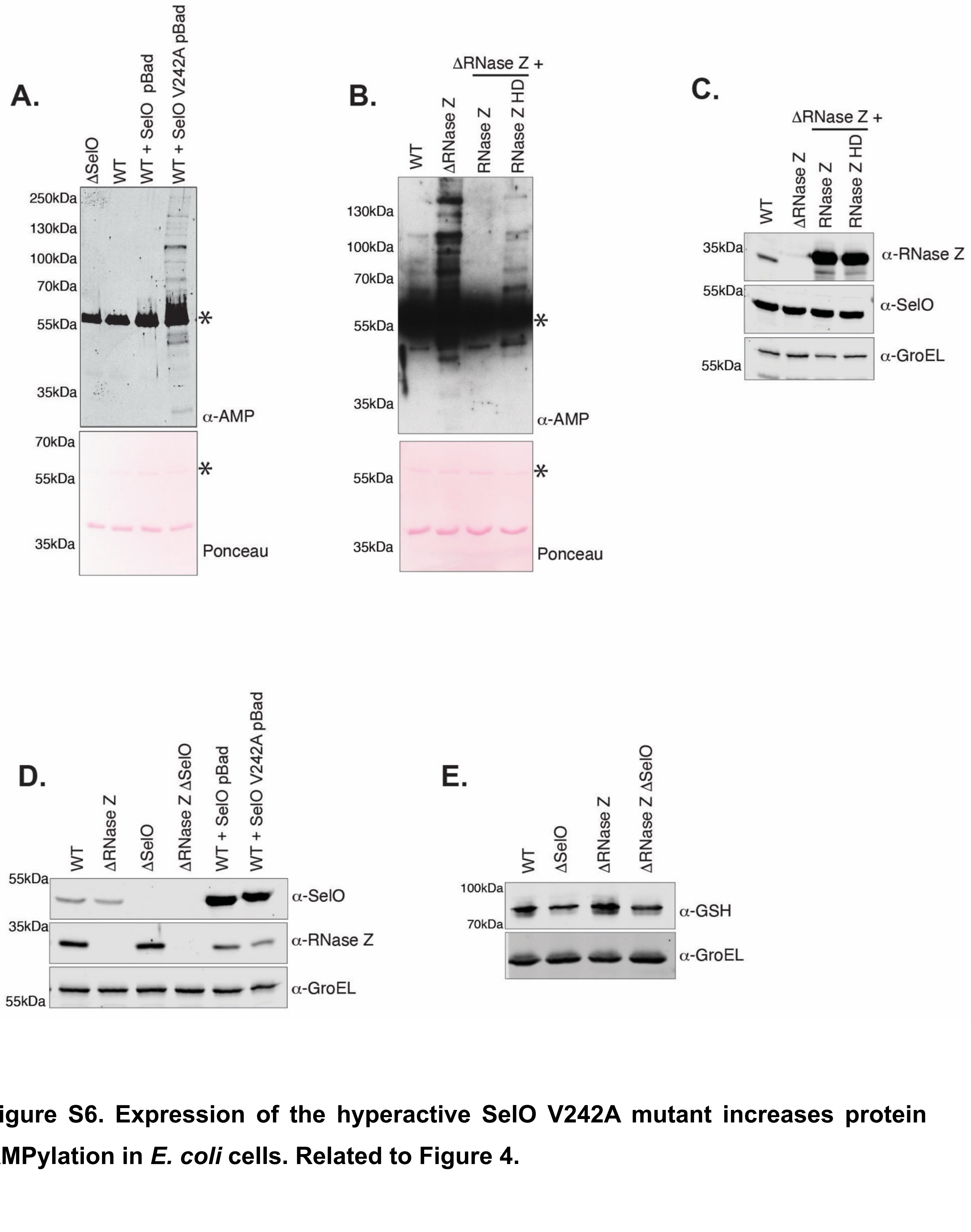

Figure S6. Expression of the hyperactive SelO V242A mutant increases protein AMPylation in *E. coli* cells. Related to Figure 4.

(A) Protein immunoblotting of AMPylated proteins enriched using GST-hinT H101N from *E. coli* BW25113 wildtype (WT), SelO knockout (ΔSelO), or *E. coli* expressing SelO or SelO V242A. The Ponceau stained membrane is shown.

(B) Protein immunoblots of AMPylated proteins enriched using GST-hinT H101N from *E. coli* wildtype (WT), RNase Z knockout (ΔRNase Z), RNase Z knockout expressing RNase Z-flag, or RNase Z knockout expressing RNase Z-flag HD. All strains are expressing SelO V242A under the endogenous SelO promoter. *denotes AMPylated GlnA.

(C) Protein immunoblots of *E. coli* extracts from *E. coli* wildtype (WT), RNase Z knockout (ΔRNase Z), RNase Z knockout expressing RNase Z-flag, or RNase Z knockout expressing RNase Z-flag HD. All strains are expressing SelO V242A under the endogenous SelO promoter. Extracts were probed for *E. coli* SelO (α-SelO), *E. coli* RNase Z (α-RNase Z) and GroEL (loading control).

(D) Protein immunoblots of *E. coli* wildtype (WT), RNase Z knockout (ΔRNase Z), SelO knockout (ΔSelO), double knockout (ΔSelOΔRNase Z), WT expressing SelO or SelO V242A under the endogenous SelO promoter.

(E) Representative protein immunoblots of *E. coli* extracts obtained from wildtype (WT), RNase Z knockout (ΔRNase Z), SelO knockout (ΔSelO), or double knockout (ΔSelOΔRNase Z) treated with diamide. Extracts were probed with anti-glutathionylation (GSH) and GroEL (loading control).

Table S1. Oligonucleotides used in this study.

Name	Sequence
gloC BamHI fwd	AAAA ggatcc ATGAACTATCGTATTATTCCG
gloC XhoI rev	aaaa CTCGAG TTACCAGACGGGCATTTC
mgtA BamHI fwd	AAAA ggatcc ATGTTTAAAGAAATTTTTACCCG
mgtA XhoI rev	aaaa CTCGAG TTATTGCCAGCCGTAAC
gloB BamHI fwd	AAAA ggatcc ATGAATCTTAACAGTATTCCC
gloB XhoI rev	aaaa CTCGAG TCAGAACCTATCTTTCTTTG
rsfS BamHI fwd	AAAA ggatcc ATGCAGGGTAAAGCACTC
rsfS XhoI rev	aaaa CTCGAG TTAACTCCAGAGTTTTTCCAG
nagD BamHI fwd	AAAA ggatcc ATGACCATTAAAAATGTAATTTGC
nagD XhoI rev	aaaa CTCGAG TCAGATAACGTCGATTTCAG
pcnB BamHI fwd	AAAA ggatcc ATGTTTACCCGAGTCG
pcnB XhoI rev	aaaa CTCGAG TCATGCGGTACCCTC
YKR070W_BamH1_F	AAAA GGATCC ATGATTGGCAAACGGTTTTTCCAAAC
YKR070W_Not1_R	AAAA GCGGCCGC TCATGCGTACTTTTCTAACGTTTTAGTAAC
YMR130W_BamH1_F	AAAA GGATCC ATGACCTATCCGAAAAGAATACCTATAAATG
YMR130W_Not1_R	AAAA GCGGCCGC TCAGGGAAAGAGTTCCTCTAAAACC
CCA1_BamH1_F	AAAAGGATCCATGCTACGGTCTACTATATCTCTACTGATG
CCA1_Not1_R	AAAAGCGGCCGCCTACAGGTATTTTGGTAGTATGGCTTTTAAATG
YBR242W_BamH1_F	AAAAGGATCCATGACAGCAACGATCACAAATAAGAAATC
YBR242W_Not1_R	AAAAGCGGCCGCTTATTTAGTTATCGATTGAGTCAAATCGGC
sidM_NdeI_F	AAAAcatatg AGCATAATGGGGAG
sidM_XhoI_R	aaaa ctc gag TTATTTTATCTTAATGGTTTGTCT
Rab1a_Bamh1_F	AAAA GGATCC g ATGTCCAGCATGAATCC
Rab1a_Sal1_R	AAAA GTCGAC TTAGCAGCAACCTCCAC
sidD 37aa BamHI fwd	AAAA GGATCC ATGCGTTCGATTATTACAC
SidD 350aa XhoI rev	aaaa CTCGAG TTA TGATAAAGCATGAATTCTTAATTG
RNaseE_aa1-529_fwd	AAAA GGATCC ATGAAAAGAATGTTAATCAACGCAACT
RNaseE_aa1-529_rev	aaaa ctc gag tta CAGCGCAGGTTGTTCC
RNaseT_BamHI_Fwd	AAAA GGATCC ATGTCCGATAACGCTCAAC
RNaseT_Xho1_rev	aaaa ctc gag TTACACCTCTTCGGCGG
E. coli RNaseZ ko fwd	CGCAATGTCACGGCAATATTGCTTAACCTGCAACATCCGACCCAGAGCGGGTGTAGGCTGGAGCTGCTTC
E. coli RNaseZ ko rev	TGACCTGCATTCACGTAACAGGTGCTGACAACCTTTGTCATCATAGCGCGATGGGAATTAGCCATGGTCC
Ec_sodA_BamHI_F	AAAAGGATCCATGAGCTATACCCTGCCATCCC
Ec_sodA_Xho1_R	AAAACTCGAGTTATTTTTTCGCCGCGAAACGTG
E. coli RNaseZ_BamH1_F	AAAAGGATCCATGGAATTAATTTTTTTAGGTACTTCAGCCG
E. coli RNaseZ_Not1_R	AAAAGCGGCCGCTTAAACGTTAAACACGGTGAAATCATTCG
E. coli RNaseZ_D68H69_f	aagccgggtaaaccaaagagagcagcgccatgaaggtgactaataa
E. coli RNaseZ_D68H69_r	ttattagtcaccttcatggcgctgctctctttggtttacccggctt

E. coli RNaseZ_d68a_f	ggtaaaccaaagagatgagcgccatgaaggtgactaa
E. coli RNaseZ_d68a_r	ttagtcaccttcatggcgctcatctctttggtttacc
E. coli RNaseZ_h69a_f	ccgggtaaaccaaagagagcatcgccatgaaggtgactaa
E. coli RNaseZ_h69a_r	ttagtcaccttcatggcgatgctctctttggtttacccgg
E. coli RNaseZ_dExoSite_fwd	gttatggctatcgtattgaagaaggtaaagcgctcgc
E. coli RNaseZ_dExoSite_rev	gcgagcgctttaccttcttcaatacgatagccataac
S. cerevisiae RNaseZ d30 EcoR1_f	aaaa GAATTC ATG TATAAATCATACAGAAAGGGACC
S. cerevisiae RNaseZ_Xho1_r	aaaa ctc gag CTAATTTTTCTTGTGTTTCTTAAGTTTGAC
S. cerevisiae RNaseZ_D544,H545A_f	cgctgattattcccaaatgggcggctgcatgcaagtgactcaga
S. cerevisiae RNaseZ_D544,H545A_r	tctgagtcacttgcatgcagccgcccatttgggaataatcagcg
H. sapiens RNaseZ_d16 bamHI Fwd	AAAA GGATCC ATGTCGCAGGGACGC
H. sapiens RNaseZ Xho1 rev	AAAA CTCGAG TCACTGGGCTCTGACC
H. sapiens RNaseZ_D550,H551A_fwd	ggcaagcccgtgtgggcagctgcgtgcaggtggg
H. sapiens RNaseZ_D550,H551A_rev	cccacctgcacgcagctgcccacacgggcttgcc
